# Human iPSC-derived 2D cortical neurons and 3D cortical organoids show fetal brain proteomic similarity and distinct phosphoproteomic profiles

**DOI:** 10.3389/fbioe.2026.1932407

**Published:** 2026-09-24

**Authors:** Rawan Albatarni, Sunkyu Choi, Kyung Chul Shin, Praveen Babu Vemulapalli, Mohammed R. Shaker, Frank Schmidt, Yongsoo Park

**Affiliations:** 1 College of Health & Life Sciences, Hamad Bin Khalifa University, Qatar Foundation, Doha, Qatar; 2 Neurological Disorders Research Center, Qatar Biomedical Research Institute, Hamad Bin Khalifa University, Qatar Foundation, Doha, Qatar; 3 Proteomics Core, Weill Cornell Medicine-Qatar, Education City, Qatar Foundation, Doha, Qatar

**Keywords:** 2D neurons, brain organoids, LC-MS/MS, phosphoproteomics, proteomics

## Abstract

Human iPSC-derived 2D cortical neurons and 3D cortical organoids (COs) are widely used to model early brain development in health and disease, but the molecular similarity of 2D and 3D models to human brain tissue at the protein and phosphorylation levels has not been systematically defined. Here, we performed paired global proteomic and phosphoproteomic profiling of 2D cortical neurons and 3D COs, and benchmarked both models against published human fetal brain, human adult frontal cortex, and mouse cortex reference datasets. Compared to mouse cerebral cortex, both 2D and 3D showed substantially higher proteomic similarity to human fetal brain. In contrast, phosphoproteomic profiles were poorly correlated between iPSC-derived models and adult human frontal cortex, and between human and mouse cortex, demonstrating that phosphorylation signaling is highly context- and developmental stage-dependent and not recapitulated by cross-species. Together, our findings establish a paired proteomic and phosphoproteomic benchmarking framework for human neuronal models, highlighting that 2D neurons and 3D COs are complementary platforms for neurodevelopmental research.

## Introduction

Human brain development is difficult to study directly, because of the ethical limitation to access developing human neural tissue, and animal models do not fully capture human-specific molecular and developmental features (Silbereis et al., 2016). Human induced pluripotent stem cell (iPSC)-derived neural models provide an important platform for studying early neurodevelopment, disease mechanisms, and therapeutic responses in a human genetic background (Brennand et al., 2011). 2D cortical neuronal cultures offer a simplified and experimentally tractable system, whereas 3D cortical organoids (COs) provide additional cellular complexity, spatial organization, and developmental architecture (Pasca, 2018). Cerebral organoids have cortical-like progenitor populations and neuronal subtypes in addition to astrocytes, supporting the utility of COs to study human brain development and neurodevelopmental disease (Lancaster et al., 2013; Sloan et al., 2018).

Advances in human pluripotent stem cell technology have enabled the generation of diverse neural cell types through increasingly refined differentiation protocols, providing scalable 2D platforms for investigating neuronal development, function, and disease mechanisms (Logan et al., 2019). More recently, 3D brain organoids have offered greater cellular diversity and tissue-like organization, while advances in multi-omics and single-cell analyses have improved the assessment of cellular identity, developmental trajectories, and disease-associated molecular changes (Logan et al., 2019; Badja et al., 2024; P and Suchitra, 2026). Nevertheless, variability in differentiation efficiency, neuronal maturation, cellular composition, and batch reproducibility remains a major challenge, emphasizing the importance of standardized protocols, rigorous quality control, and complementary molecular and functional validation and characterization.

Although transcriptomic profiling has been widely used to characterize iPSC-derived neuronal models, protein-level characterization remains essential, because protein abundance more directly reflects cellular function, pathway activity, and disease-relevant molecular phenotypes (Liu et al., 2016). Large-scale human tissue proteome studies, such as the human proteome map by Kim et al., provide valuable reference datasets for benchmarking *in vitro* neural models against fetal brain, adult brain, and non-neural tissues (Carlyle et al., 2017; Kim et al., 2014; McKetney et al., 2019). However, few studies have systematically compared 2D and 3D human neuronal models at the global proteome/phosphoproteome level and directly evaluated their similarity to human and mouse brain reference proteomes.

Phosphorylation represents a critical regulatory layer controlling neuronal signaling, synaptic function, cytoskeletal dynamics, and developmental pathway activity (Cohen, 2002; Trinidad et al., 2012). Phosphoproteomics is particularly informative, because phosphorylation can change rapidly in response to cellular state, kinase and phosphatase activity, developmental stage, extracellular stimulation, and disease conditions (Lemeer and Heck, 2009). Previous large-scale phosphoproteomic studies have shown that phosphorylation patterns are highly tissue-specific and can vary independently of total protein expression (Huttlin et al., 2010; Ping et al., 2020). Therefore, phosphoproteomic profiling may reveal model-specific signaling states, which are unique from global proteomic analysis.

In this study, we performed paired global proteomic and phosphoproteomic profiling of human 2D cortical neurons (56-Day of differentiation) and 3D COs (110-Day of differentiation). First, we confirmed functionally active human COs using microelectrode array (MEA) recording. We then compared the global proteomes and phosphoproteomes of 2D and 3D models. Finally, we benchmarked 2D and 3D datasets against published human fetal brain, adult cortex, mouse cortex, and reference phosphoproteomic datasets. Our study provides paired protein- and phosphoprotein-level comparisons of human 2D and 3D neuronal models against both human and mouse brain references, highlighting context- and developmental stage-dependent divergence in phosphoproteomic signaling.

## Methods

### Differentiation of 2D cortical neurons

IMR90 iPSC lines (WiCell Research Institute) were used for 2D and 3D models. Control iPSCs were differentiated into cortical neurons following the published protocols (Ali et al., 2023). To initiate differentiation, iPSC colonies were dissociated into single cells using TrypLE and plated onto Matrigel-coated plates in mTeSR1 medium containing 10 μM Y-27632 (ROCK inhibitor, STEMCELL Technologies, cat# 72307). Next day, the cells were 90%–100% confluent and differentiation was initiated by changing medium to Neurobasal medium (DMEM/F12, Neurobasal, 1X B-27 minus vitamin A, 1X N2 supplement, 1X L-Glutamine, 1X Non-essential amino acids (NEAA), 50 μM β-mercapto-ethanol, 0.4X Penicillin/streptomycin) supplemented with 10 μM SB431542 (Tocris, cat# 1614) and 2 μM Dorsomorphin (Tocris, cat# 3093) for 12 days. During neural induction, the cells were split on day 7 and 14 using TrypLE and plated onto Matrigel-coated plates in neurobasal media containing 10 μM Rock inhibitor. For neural progenitor proliferation (days 14–18), the neurobasal media was supplemented with 20 ng/mL bFGF. At day 20, neural progenitor cells (NPCs) were cryopreserved or plated for maturation onto Matrigel-coated plates in neurobasal media supplemented with 10 ng/mL BDNF, 10 ng/mL GDNF, 20 μM dibutyryl-cyclic AMP (db-cAMP, Sigma), and 200 μM Ascorbic acid (AA, Sigma). At day 28, the cells were plated for experiments onto 100 μg/mL poly-L-ornithine (PO, Sigma, cat# P8638) and 20 μg/mL laminin (Invitrogen, cat# 23017015)-coated plates and media was changed every 2–3 days. We previously reported that cortical neurons become matured after 6–8 weeks of differentiation (Shin et al., 2023). 2D cortical neurons at 56-Day (8-week) of differentiation were used for proteomics. The three 2D samples were generated from independent differentiation experiments.

### Generation of 3D cortical brain organoids

Control iPSCs were differentiated into cortical brain organoids as previously described (Sloan et al., 2018), with minor modifications. Briefly, iPSCs were dissociated into single cells using TrypLE and seeded into AggreWell™800 plates (STEMCELL Technologies, cat# 34825) at a density of 300,000 cells per well in mTeSR1 medium supplemented with 10 μM Y-27632. Plates were centrifuged at 800 rpm for 5 min at room temperature to facilitate cell aggregation at the bottom of the microwells. The following day, spheroids were collected and washed to remove Y-27632. Spheroids were then gently resuspended and transferred to ultra-low attachment plates containing DMEM/F12 basal medium composed of DMEM/F12, 20% KnockOut™ Serum Replacement (Gibco, #10828-010), 1× non-essential amino acids (NEAA), 1× L-glutamine, 0.4× penicillin/streptomycin, and 100 μM β-mercaptoethanol. The medium was supplemented with 10 μM SB431542 (Tocris, #1614) and 2 μM dorsomorphin (Tocris, #3093). Medium was changed daily for 4 days, and plates were placed on an orbital shaker from day 3 onward.

On day 5, the medium was replaced with a 1:1 mixture of DMEM/F12 basal medium and Neurobasal medium containing DMEM/F12, Neurobasal, 1× B-27 supplement without vitamin A, 1× N2 supplement, 1× L-glutamine, 1× NEAA, 50 μM β-mercaptoethanol, and 0.4× penicillin/streptomycin. On day 6, the medium was replaced with complete Neurobasal medium supplemented with 20 ng/mL epidermal growth factor (EGF) and 20 ng/mL basic fibroblast growth factor (bFGF). Medium was exchanged daily until day 15 and every 2 days thereafter. On day 26, EGF and bFGF were withdrawn, and the Neurobasal medium was supplemented with 10 ng/mL brain-derived neurotrophic factor (BDNF), 10 ng/mL glial cell line-derived neurotrophic factor (GDNF), 20 μM dibutyryl-cyclic AMP (db-cAMP; Sigma), 200 μM ascorbic acid (Sigma), and 1% Matrigel. Matrigel supplementation was discontinued on day 28. On day 43, BDNF, GDNF, db-cAMP, and ascorbic acid were withdrawn. Organoids were maintained and cultured for up to 110 days 3D organoids at 110-Day differentiation were used for proteomics. The organoid samples were generated in a single differentiation experiment and maintained in separate culture wells, with individual organoids analyzed as experimental replicates.

### Multi-well microelectrode array (MEA) analysis

24-well MEA plates were coated with poly-L-ornithine and laminin and brain COs were plated in the center of the well; one organoid per well. Extracellular recordings from organoids were performed 1 week after plating using the Maestro Pro platform (Axion Biosystems). Each well contains a 4 × 4 16 channel electrode array with four reference electrodes. Extracellular voltage recordings were collected at a sampling rate of 12.5 kHz per channel. A band-pass filter (200 Hz–3 kHz cut-off frequencies) was applied with a variable threshold spike detector at ±6 standard deviations of the root mean square (RMS) of the background noise. During recording, the MEA plate was maintained at 37 °C and 5% CO_2_. MEA data analysis was performed using the Axion Biosystems Neural Metric Tool (Axion Biosystems). An electrode was considered active at a threshold of 3 spikes/min. Network Bursts were defined as at least 5 consecutive spikes across multiple electrodes with interspike intervals (ISI) of less than 100 ms and a minimum of 10% electrodes. Baseline, TTX, and washout MEA measurements were obtained sequentially from the same organoids and were therefore analyzed as paired measurements. A repeated-measures one-way ANOVA was used to compare the paired measurements.

### Sample preparation for liquid chromatography–tandem mass spectrometry (LC–MS/MS)

2D and 3D samples were processed using the PreOmics iST kit (PreOmics GmbH, Martinsried, Germany) as previously described (Kulak et al., 2014; Niu and Mann, 2019). Briefly, 100 µg of each protein sample were transferred into a 96-well plate using an Agilent Bravo automated liquid handling system. Each sample was adjusted to a final volume of 50 µL with LYSE buffer to promote protein solubilization, reduction, and alkylation, followed by incubation at 95 °C for 10 min. For enzymatic digestion, 50 µL of resuspended DIGEST buffer was added to each well, and samples were incubated at 37 °C for 3 h. Digestion was terminated by the addition of 100 µL STOP buffer, after which the peptide mixtures were transferred to cleanup cartridges. Peptides were purified by sequential washing with 200 µL WASH buffer 1 and 200 µL WASH buffer 2. Bound peptides were eluted twice with 100 µL ELUTE buffer to maximize peptide recovery. The combined eluates were dried using a vacuum concentrator and stored at −80 °C until LC–MS/MS analysis.

Phosphopeptide enrichment was performed on the AssayMAP Bravo platform using iron-nitrilotriacetate (Fe(III)-NTA) cartridges. Cartridges were first primed with 250 µL priming buffer containing 50% acetonitrile and 0.1% trifluoroacetic acid (TFA) at a flow rate of 300 μL/min. Cartridges were then equilibrated with 200 µL loading buffer containing 80% acetonitrile and 0.1% TFA at 10 μL/min. Peptide samples were loaded onto the equilibrated Fe(III)-NTA cartridges at 1.0 μL/min to maximize phosphopeptide binding. Non-specifically bound peptides were removed by washing the cartridges with 250 µL wash buffer containing 80% acetonitrile and 0.1% TFA at 10 μL/min. Bound phosphopeptides were eluted with 25 µL of 2% ammonium hydroxide at 2 μL/min and immediately neutralized with 25 µL of 10% formic acid. The resulting phosphopeptide-enriched eluates were dried using a vacuum concentrator and stored at −80 °C until LC–MS/MS analysis.

### LC–MS/MS data acquisition and analysis

Global proteomic analysis was performed using a timsTOF HT mass spectrometer (Bruker) coupled to an Evosep One LC system operated with the 30SPD 44-min standard gradient on an EV1109 Performance column. Peptides were acquired in data-independent acquisition (DIA) mode using diaPASEF, with 32 MS/MS windows per cycle covering 400–1201 m/z and an ion mobility range of 0.60–1.60 1/K_0_. Raw DIA data were processed in Spectronaut 20 (Biognosys) using direct DIA analysis against the *Homo sapiens* UniProtKB database (June 2025 release). Carbamidomethylation of cysteine was set as a fixed modification, while N-terminal acetylation and methionine oxidation were set as variable modifications. Label-free quantification (LFQ) was performed, and only proteins identified with at least two unique peptides were retained for downstream analysis. Raw protein intensities were log_2_-transformed and median-centered within each replicate by subtracting the median intensity of that replicate to reduce inter-run variation and loading differences. Proteins with missing values across all replicates within a given condition were excluded from the corresponding analysis; detected in more than 2 of 3 replicates.

For phosphoproteomic analysis, enriched phosphopeptides were analyzed using a Q Exactive Exploris 480 mass spectrometer (Thermo Fisher Scientific) coupled to a Vanquish Neo UHPLC system in data-dependent acquisition (DDA) mode. Peptides were trapped on an Acclaim PepMap 100 Nano-Trap column and separated on an EASY-Spray PepMap Neo UHPLC C18 column using a 180-min acetonitrile gradient. Full MS scans were acquired over 400–1650 m/z at 120,000 resolution, followed by HCD-MS/MS fragmentation at 30,000 resolution with 28% normalized collision energy. Raw phosphoproteomic data were processed using SpectroMine (Biognosys) against the *Homo sapiens* UniProtKB database (June 2025 release). Carbamidomethylation of cysteine was set as a fixed modification, while N-terminal acetylation, methionine oxidation, and phosphorylation of serine, threonine, and tyrosine were set as variable modifications. Phosphopeptides were identified and filtered based on a localization probability threshold of 0.75 (SpectroMine default). This approach focuses on Class I sites, providing stringent confidence in the site-specific assignment of phosphorylation. Zero intensity values were treated as missing values before log_2_ transformation. For peptide-level analyses, phosphopeptide intensities were log_2_-transformed and median-centered within each replicate. For gene-level phosphoproteomic analyses, phosphopeptides assigned to the same gene symbol were summed within each replicate to preserve the total phosphorylation signal associated with each gene. The resulting gene-level phospho-intensity matrix was then log_2_-transformed, median-centered per replicate, and averaged across biological replicates to obtain a single gene-level phospho-abundance value for each condition.

Proteins were quantified by label-free quantification (MaxLFQ) using Spectronaut. 2D cortical neurons (n = 3 replicates) and 3D brain organoids (n = 3 replicates) were jointly processed in a single MaxLFQ run to place both models on a common intensity scale. This yielded 10,017 protein groups with valid quantification in all six samples. Protein intensities were log_2_-transformed and median-centered per replicate before all downstream analyses.

Phosphopeptides were quantified by label-free quantification using SpectroMine, with 2D and 3D samples processed jointly to place both models on a common intensity scale. Phosphopeptides with valid intensities in ≥2 replicates were retained (2D: n = 2 replicates; 3D: n = 3 replicates).

### Differential abundance analysis

To identify proteins differentially abundant between 2D neurons and 3D organoids, a Welch’s t-test was applied to the log_2_ median-centered intensities of all 10,017 protein groups. P-values were corrected for multiple testing using the Benjamini–Hochberg (BH) method. Proteins with |log_2_FC| ≥ 0.585 (≥1.5-fold change) and FDR <0.05 were considered significantly different. log_2_FC is defined as mean (3D) − mean (2D). This identified 1,973 proteins with higher abundance in 3D organoids and 707 proteins with higher abundance in 2D neurons.

For phosphoproteomics, phosphopeptides were first aggregated to gene level by computing the mean log_2_ LFQ intensity across all phosphopeptides per gene per replicate. Genes with complete data across all five replicates of 2D and 3D were retained (n = 2,609 phosphoproteins). A Welch’s t-test was then applied. Because the 2D condition has only n = 2 replicates, BH multiple-testing correction yields no significant results; a nominal p < 0.05 threshold was therefore applied without FDR correction.

### Principal component analysis

PCA was performed on the 10,017 × 6 log_2_ median-centered intensity matrix. Protein values were z-scored across samples prior to decomposition (scikit-learn). PC1 explained 60.3% of total variance and cleanly separated 2D neurons from 3D organoids; within-model replicates clustered tightly.

### Proteome correlation

Pearson and Spearman correlations between the two models were computed on mean log_2_ LFQ abundances across the 10,017 shared proteins (proteome: r = 0.741) and on mean log_2_ LFQ abundances across 6,461 phosphopeptides detected in both models with ≥2 valid replicates per model (phosphopeptides: r = 0.597 and phosphoproteins: r = 0.552). Per-sample Pearson r was also computed across all six samples to assess replicate reproducibility (within-model r > 0.92 for both 2D and 3D).

### Functional group and GO enrichment analysis

Differentially abundant proteins were mapped to curated gene sets covering cell-type markers (CellMarker 2.0/PanglaoDB), synaptic compartments (SynGO), ion channels, mitochondria/OXPHOS, cytoskeleton, neurotransmitter synthesis, proteasome/UPS, chaperones/HSP, mTOR/PI3K signaling, ASD/NDD risk genes, and ECM/matrisome. The percentage of each gene set represented among the 2D-higher and 3D-higher protein groups was computed. Gene Ontology and pathway enrichment was performed using g:Profiler (organism: *Homo sapiens*; sources: GO:BP, GO:MF, GO:CC, KEGG, Reactome; FDR <0.05 via g:SCS correction; background: all 9,874 detected genes).

### Cross-tissue benchmarking

To assess the proteome identity of both iPSC models, mean log_2_ LFQ per gene was correlated against human tissue proteomes from Kim et al. (2014) (E-PROT-1), which covers 30 tissues including fetal brain and adult heart (Kim et al., 2014). Gene-level means were obtained by averaging log_2_ intensities across replicates, then across protein groups sharing the same gene symbol. Reference abundances were log_2_-transformed and median-centered before correlation. Both models were further benchmarked against five independent human brain proteomics studies (Carlyle et al., 2017; Kim et al., 2014; McKetney et al., 2019) (E-PROT-1 for Kim_2014; E-PROT-30 for Carlyle_2017; E-PROT-61 for McKetney_2019, control samples were used for reference comparisons) and against mouse cerebral cortex (Geiger 2013, E-PROT-11; mouse–human orthologues from HomoloGene/mygene. info) (Geiger et al., 2013) to contextualise their brain proteome correlation within known inter-study and cross-species variation.

### Differential phosphoproteomics

Gene-level phosphoprotein abundance was computed by log_2_-transforming phosphopeptide LFQ intensities (replacing zeros with missing), median-centring each replicate, and averaging across peptides per gene per replicate, then across replicates per model. A Welch’s t-test was applied to all 2,609 gene-level phosphoproteins detected in both models. Because the 2D condition has only n = 2 replicates, a nominal p < 0.05 threshold was therefore applied without FDR correction. Significance was additionally required to satisfy |log_2_FC| ≥ 0.585. This identified 226 phosphoproteins with higher abundance in 2D neurons and 122 with higher abundance in 3D organoids.

Twenty curated signaling pathway gene sets were assembled for pathways relevant to neural function (MAPK/ERK, PI3K/AKT, mTOR, CDK, PKA/CREB, PKC, CaMKII, Wnt, Hippo/YAP, Notch, BMP/TGFβ, BDNF/TrkB, GSK3/Tau, AMPK, p38/JNK, Src/FAK, ErbB/EGFR, JAK/STAT, DNA damage response, synaptic plasticity). For each gene set, the number and percentage of members detected among the 2,609 phosphoproteins was computed, and the fraction with significantly higher phosphorylation in 2D or 3D (nominal p < 0.05, |log_2_FC| ≥ 0.585) was reported. Gene Ontology and pathway enrichment for 2D-higher and 3D-higher phosphoprotein sets was additionally tested using g:Profiler.

### Kinase-substrate enrichment analysis (KSEA)

Kinase activity was inferred from gene-level phosphoprotein log_2_FCs using a z-score-based KSEA approach (Casado et al., 2013). Kinase–substrate relationships were retrieved from OmniPath (cached locally; 39,015 substrate entries, 1,648 kinases). For each kinase with ≥3 detected substrates, a z-score was computed as:

$$z=\left(\bar{m}\_s-\bar{m}\_\text{bg}\right)/\left(\sigma \_\text{bg }/\;\sqrt{n}\right)$$

where m̄_s is the mean log_2_FC of the kinase’s substrates, m̄_bg is the mean log_2_FC of all background phosphoproteins, σ_bg is the background standard deviation, and n is the number of detected substrates. Kinases with nominal p < 0.05 (two-sided z-test) were considered significant. This analysis used all 2,609 detected phosphoprotein log_2_FCs as the ranked input; no significance pre-filter was applied.

### Kinase library motif enrichment

Kinase recognition motif enrichment was performed using the Kinase Library (Johnson et al., 2023). Phosphopeptides were tested at the individual site level. A peptide-level Welch’s t-test was first applied to all phosphopeptides passing the ≥2/2 2D and ≥2/3 3D validity filter (n = 10,450 peptides tested), yielding 866 peptides significantly higher in 3D (UP: log_2_FC > 0.585, nominal p < 0.05) and 819 significantly higher in 2D (DOWN). Phosphosite sequences were extracted as 15-mer windows centred on the modified residue using the Spectronaut Phospho (STY) probability annotations (localisation probability ≥0.75 required). For each kinase and each direction (UP/DOWN), a Fisher’s exact test was applied to ask whether the kinase’s high-scoring sites (percentile ≥75 of the kinase’s score distribution) are over-represented in that direction. P-values were adjusted by Benjamini–Hochberg correction across kinases within each amino acid class (Ser/Thr and Tyr separately). Kinases with adjusted p < 0.05 were reported as enriched.

### Integration of phosphoproteomics and global proteomics

To distinguish protein-level abundance changes from phosphorylation stoichiometry changes, protein-level phospho log_2_FCs (nominal p < 0.05 threshold) were merged with protein log_2_FCs (FDR <0.05 threshold) for all 2,449 proteins detected in both the phosphoproteomics and proteomics datasets. Proteins were classified into four categories. 1) Phospho-specific (n = 233): phospho significant, protein not significant—reflects true changes in phosphorylation stoichiometry or kinase/phosphatase activity. 2) Protein-specific (n = 555): protein significant, phospho not significant—reflects bulk abundance changes not accompanied by detectable phosphorylation changes. 3) Concordant (n = 54): both significant, same direction—phospho change likely driven by protein level change. 4) Discordant (n = 40): both significant, opposite direction—phospho change opposes the protein level trend. The remaining 1,567 genes were classified as background (neither significant). Results were visualised as a scatter plot (protein log_2_FC × phospho log_2_FC) with a companion strip chart of the phospho-specific hits sorted by phospho log_2_FC.

### Phosphoproteome benchmarking

2D and 3D phosphoproteomes were compared with mouse brain phosphoproteome. Mean protein-level log_2_ phosphoprotein abundance for each 2D and 3D models was correlated with the mouse whole-brain phosphoproteome from Huttlin et al. (2010) (Huttlin et al., 2010), corresponding to Expression Atlas accession E-PROT-13. Mouse iBAQ phosphoprotein values were log_2_-transformed and median-centered; mouse gene symbols were mapped to human orthologues using NCBI HomoloGene/mygene. info (5,112 unique orthologous human gene symbols). Pearson and Spearman correlations were computed on all genes detected in both datasets. 2D vs. mouse brain phospho: r = 0.156 (n = 700); 3D vs. mouse brain phospho: r = 0.169 (n = 780). For contextualisation, cross-species regular proteomics correlations (2D and 3D vs. mouse cerebral cortex, Geiger 2013 E-PROT-11) were also computed and shown alongside: 2D vs. mouse cortex proteome r = 0.534 (n = 2,566); 3D vs. mouse cortex proteome r = 0.531 (n = 2,346), consistent with previously reported cross-species proteomics benchmarks.

2D and 3D phosphoproteomes were compared with human frontal cortex phosphoproteome. Mean gene-level log_2_ phosphoprotein abundance was correlated with the human frontal cortex phosphoproteome from the Seyfried laboratory (Ping et al., 2020) (syn20820053; frontal cortex BA9, TMT6plex, n = 10 normal controls; BA = Brodmann area). Human phosphoproteome values are TMT log_2_ ratios (sample/batch reference channel 126) averaged across all phosphopeptides per gene, then across the 10 CTL replicates, and median-centered. Results: 2D vs. human cortex phospho: r = 0.036 (n = 547); 3D vs. human cortex phospho: r = 0.032 (n = 617). The near-zero correlation reflects the methodological disparity between TMT ratio quantification (narrow dynamic range, −1.6 to +0.5) and LFQ intensity (wide dynamic range), as well as the expected biological divergence between immature iPSC-derived neurons and adult human cortex. Pearson r is invariant to the median-centering applied to both datasets.

Mouse brain phosphoproteome was compared with human frontal cortex phosphoproteome. To establish a cross-species phosphoproteomics baseline independent of the iPSC-derived 2D and 3D proteomes data, the mouse whole-brain phosphoproteome (E-PROT-13, ortholog-translated) was directly correlated with the human frontal cortex phosphoproteome (syn20820053). Both series were log_2_-transformed and median-centered as described above. A total of 1,223 genes were detected in both datasets. The cross-species correlation was Pearson r = 0.226 (p = 1.4 × 10^−15^) and Spearman ρ = 0.267 (p = 2.0 × 10^−21^).

### Statistical analysis and software

All analyses were performed in Python 3.12.3 using the following packages: pandas 3.0.2 for data manipulation, NumPy 2.4.4 for numerical operations, SciPy 1.17.1 for correlation and statistical testing, statsmodels 0.14.6 for Benjamini–Hochberg FDR correction. Figures, including volcano plots, scatter plots, and correlation matrices were generated using Matplotlib 3.10.8 and GraphPad Prism 9 (GraphPad Software, San Diego, CA, USA). Spearman correlation was used as a non-parametric complement to Pearson correlation where indicated. Bioinformatic analyses were performed with the assistance of Claude Code (Anthropic) within Visual Studio Code (Microsoft).

## Results

### Generation and functional validation of human cortical organoids

To enable direct proteomic and phosphoproteomic comparison between 2D and 3D human neural models, we generated 3D cortical organoids (COs) from control human iPSCs using a stepwise differentiation protocol comprising neural induction, neural progenitor expansion, and cortical neuronal maturation (Figure 1a). The 2D cortical neuronal model used for comparison was generated as previously described, and has been shown to produce functionally active glutamatergic cortical neurons (Shin et al., 2023). We have previously demonstrated that 3D COs generated using this protocol express cortical neuronal markers BRN2 and CTIP2, as well as the neural progenitor marker SOX2 (Ali Moussa et al., 2022).

**FIGURE 1:**
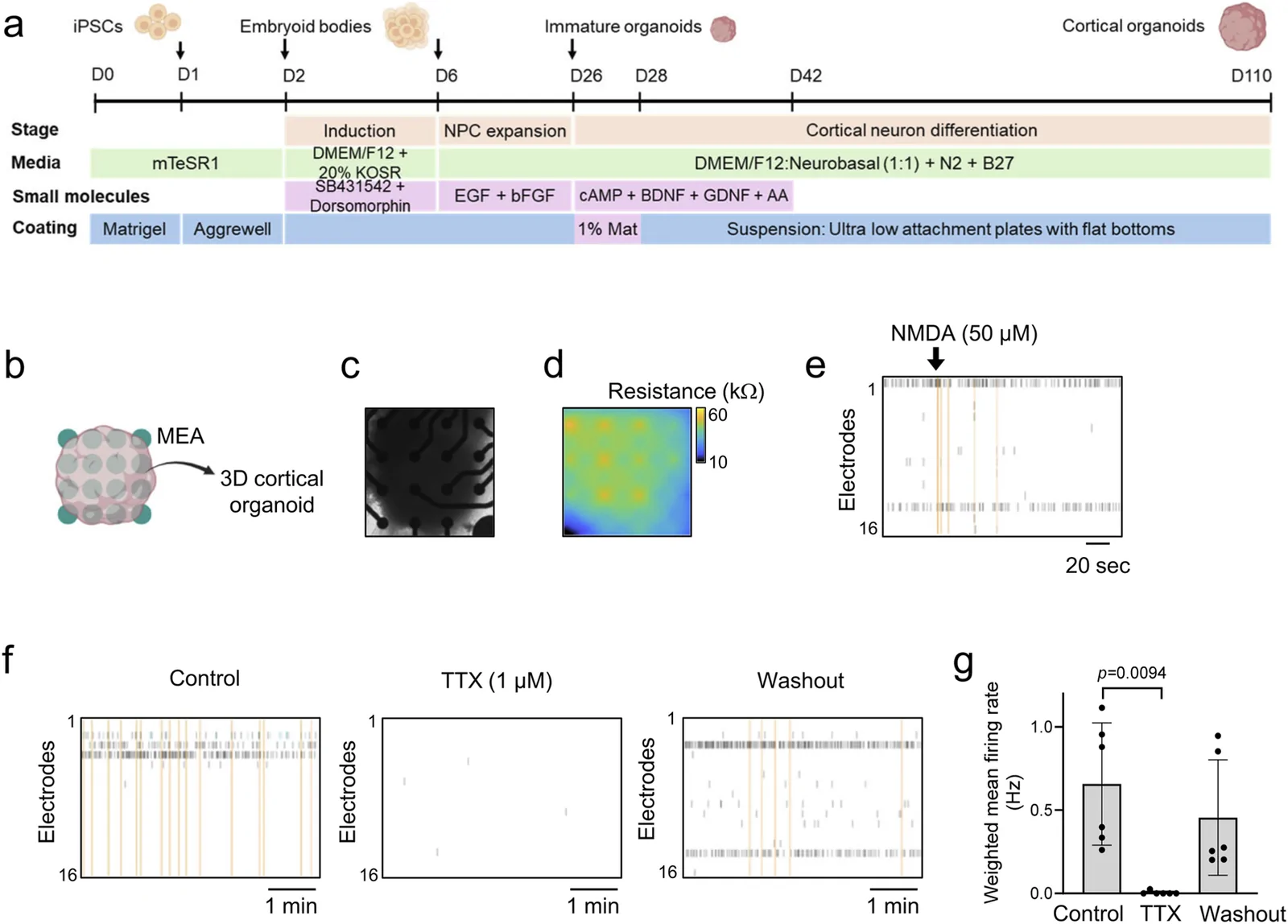
Generation of human brain cortical organoids (COs) and functional validation using MEA recording. **(a)** Schematic workflow for the generation of human COs from human pluripotent stem cells. Cells were subjected to neural induction, neural progenitor cell expansion, and cortical neuronal differentiation using defined media conditions, extracellular matrix substrates, and stage-specific supplements. **(b)** Schematic illustration of a CO positioned on the electrodes of a microelectrode array (MEA) plate for extracellular electrophysiological recording. **(c)** Representative image showing a cortical organoid placed on the MEA electrode field. **(d)** Representative viability image demonstrating that the CO remained attached to the electrode area during recording. **(e)** Representative MEA raster plot showing spontaneous and evoked neuronal activity following NMDA application, indicating functional excitatory responsiveness of CO. **(f)** Representative MEA raster plots before, during, and after tetrodotoxin (TTX) treatment. TTX completely inhibited neuronal firing activity, and activity was restored after washout. **(g)** Quantification of weighted mean firing rate across conditions. Data represent mean ± SEM from individual organoids (n = 6). A repeated-measures one-way ANOVA was used to compare the paired measurements.

To assess functional neuronal activity in the 3D model, mature COs generated using our published protocol (Shaker et al., 2021) were positioned directly onto the electrodes of MEA plates for extracellular recording of spontaneous and pharmacologically evoked network activity (Figures 1b–d). Viability imaging based on the electrode resistance confirmed stable attachment of COs to the electrode area throughout recording (Figure 1d). MEA recordings revealed spontaneous neuronal firing in COs, and NMDA treatment further increased spiking activity, demonstrating the presence of functionally responsive excitatory neuronal networks (Figure 1e). To confirm that recorded signals were driven by neuronal action potential-dependent activity, COs were treated with tetrodotoxin (TTX), a voltage-gated sodium channel blocker. TTX completely abolished firing, and subsequent washout restored neuronal activity, action potential-dependent MEA signals (Figures 1f,g). As reported in our group (Shin et al., 2023), 2D cortical neurons are functionally active, generating neural network activity mediated by mainly glutamatergic excitatory neurons. Together, the 3D COs are functionally active and suitable for downstream proteomic and phosphoproteomic analyses.

### Global proteomic comparison of 2D neurons and 3D cortical organoids

To define the molecular landscape of 2D cortical neurons (56-Day differentiation) and 3D COs (110-Day differentiation), we performed LC-MS/MS-based global proteomic profiling using data-independent acquisition (DIA) (Figure 2a). Pearson correlation analysis of proteins (10,017 proteins) showed a positive but incomplete relationship between 2D and 3D proteomes (r = 0.741; Figure 2b), indicating substantial proteomic divergence between the two systems. Volcano plot analysis confirmed differentially abundant proteins enriched in each model (Figure 2c); 707 proteins enriched in 2D (blue) and 1,973 proteins enriched in 3D (red). PCA analysis revealed clear separation between the 2D neuron and 3D CO proteomes, indicating distinct global protein abundance profiles between 2D and 3D models (Supplementary Figure S1a). Within each model, biological replicates showed high reproducibility, with pairwise Pearson correlations exceeding r = 0.90 for both 2D neurons and 3D COs, whereas cross-model correlations were consistently lower, at r ≈ 0.70, further supporting model-specific proteomic differences (Supplementary Figure S1b).

**FIGURE 2:**
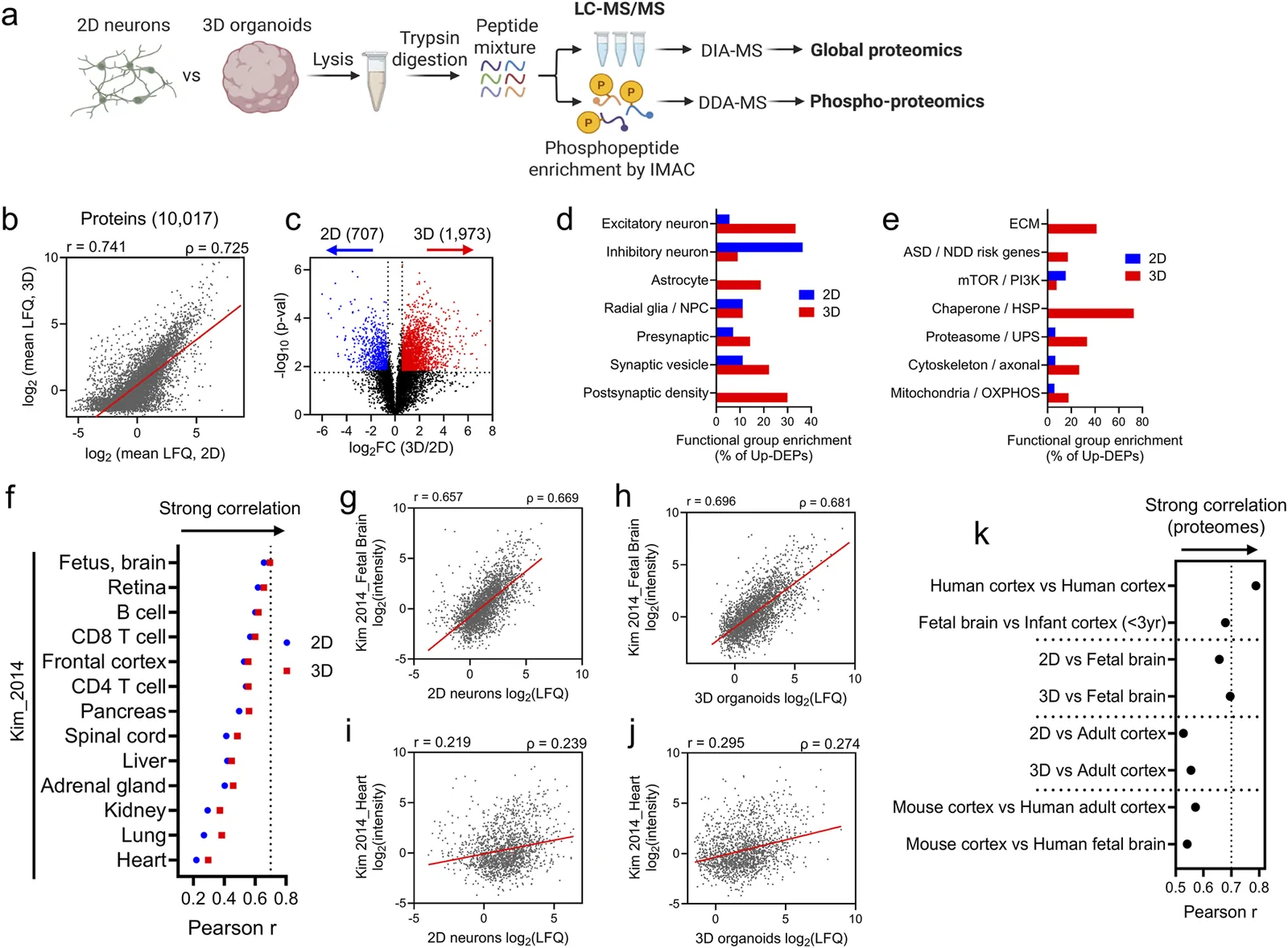
Global proteomic profiling of 2D neurons and 3D COs and comparison with reference human brain proteomes. **(a)** Schematic overview of the LC-MS/MS workflow for global proteomic and phosphoproteomic analyses. For global proteomics, peptides were analyzed by DIA-MS, whereas phosphopeptides were enriched by immobilized metal affinity chromatography (IMAC) and analyzed by DDA-MS for phosphoproteomic profiling. **(b)** Scatter plot showing the correlation of protein abundance between 2D neurons and 3D cortical organoids for commonly identified proteins. **(c)** Volcano plot showing differentially expressed proteins between 2D and 3D. Proteins enriched in 2D and 3D are indicated in blue and red, respectively. **(d,e)** Functional enrichment analysis of proteins enriched in 2D neurons and 3D cortical organoids. **(f)** Pearson correlation coefficients (r) comparing our 2D and 3D proteomes with tissue proteomes from the published human proteome dataset of Kim et al., 2014. Fetal brain and heart showed the highest and least similarity among reference tissues, respectively. **(g ,h)** Scatter plots showing the correlation between the fetal brain proteome and our 2D **(g)** or 3D proteome **(h)**. **(i,j)** Scatter plots showing the correlation between the human heart proteome and our 2D **(i)** or 3D proteome **(j)**. **(k)** Summary plot of Pearson’s correlation coefficients (r) comparing our 2D and 3D proteomes with published reference proteomes. Reference datasets include human fetal brain and human adult cortex proteomes, with additional comparison to mouse cortex where indicated. Higher r values indicate greater proteomic similarity.

Functional enrichment analysis showed distinct molecular features associated with 2D neurons and 3D cortical organoids. Proteins enriched in 3D organoids were associated with excitatory neuronal markers, synaptic vesicles, post-synaptic density, extracellular matrix (ECM) organization, protein folding and proteostasis, protein degradation pathways, and synaptic maturation, suggesting increased structural complexity and higher cellular remodeling in the 3D model (Figures 2d,e). Notably, proteins related to calcium signaling and neuronal excitability were enriched in 3D organoids. In contrast, 2D neurons showed enrichment of proteins associated with inhibitory neuronal markers, dopamine and serotonin biosynthetic enzymes, and modestly increased mTOR-related signaling. Although both systems retained core neural proteomic signatures, the 3D organoids showed broader functional diversity, with 44 enriched Gene Ontology (GO) terms compared with 12 terms in 2D neurons (Supplementary Figures S1c,d). Interestingly, 3D COs showed enrichment of proteins associated with exosomes and extracellular vesicles (EVs), consistent with our previous report that brain COs release predominantly NCAM-positive, neuron-derived EVs (Ali Moussa et al., 2022), suggesting that 3D COs provide a useful platform for studying EV-mediated intercellular communication in neurodevelopmental disease models. Together, these findings suggest that while both models preserve neural proteomic identity, the model-specific protein populations reflect distinct molecular states: 2D neurons exhibit features consistent with monolayer neuronal culture, whereas 3D organoids display a more complex and developmentally organized proteomic landscape.

Next, to benchmark both models against human tissue proteomes, we compared the protein abundance profiles of 2D and 3D models with the multi-tissue human proteome atlas reported by Kim et al. (2014) (Kim et al., 2014), which includes fetal brain, adult frontal cortex, and adult heart (Figure 2f). Fetal brain showed the strongest proteomic similarity to both 2D neurons and 3D COs, whereas adult heart showed the weakest correlation, consistent with the neural identity of 2D and 3D *in vitro* models. Scatter plot comparisons confirmed positive correlations between fetal brain and both 2D and 3D proteomes (r ≈ 0.70; Figures 2g,h), whereas human heart proteome showed minimal correlation (r = 0.2–0.3; Figures 2i,j).

To contextualize our proteomics data against inter-study variability in human brain proteomes, we calculated pairwise Pearson correlations across independent adult cortex and fetal/infant brain reference datasets (Carlyle et al., 2017; Kim et al., 2014; McKetney et al., 2019) (Figure 2k). Inter-study correlations among adult cortex datasets were high (r ≈ 0.80), while fetal and infant brain datasets showed greater inter-study variability (r ≈ 0.70), reflecting the greater biological heterogeneity of the developing brain across donors and developmental stages. Notably, both 2D and 3D models aligned most closely with fetal brain proteomes, with correlation values comparable to those observed across independent fetal brain studies. In contrast, mouse cerebral cortex proteome showed lower correlation with human adult cortex or human fetal brain (r ≈ 0.55). Together, 2D cortical neurons and 3D COs capture fetal brain-like proteomic features, supporting 2D and 3D as human neurodevelopmental models to investigate neurodevelopmental disorders.

The 2D cultures and 3D organoids were analyzed at protocol-specific rather than age-matched differentiation endpoints. Therefore, the observed molecular differences between 2D and 3D models represent the combined effects of culture duration, developmental stage, cellular composition, differentiation protocol, and dimensionality. Despite these differences, both models showed the strongest proteomic similarity to the human fetal cortex, supporting complementary utility of 2D and 3D cultures for modeling early human cortical development.

### Comparative phosphoproteomic profiling of 2D neurons and 3D cortical organoids

To determine whether 2D neurons and 3D cortical organoids differ in phosphorylation-dependent signaling independently of total protein abundance, we performed phosphoproteomic profiling using Fe(III)-NTA-based phosphopeptide enrichment followed by data-dependent acquisition (DDA) LC-MS/MS. This analysis identified 8,546 phosphopeptides in 2D neurons and 10,795 phosphopeptides in 3D cortical organoids, of which 6,462 phosphopeptides were shared between the two models. In addition, 2,084 phosphopeptides were unique to 2D neurons, whereas 4,333 phosphopeptides were unique to 3D organoids, indicating broader phosphoproteomic coverage and condition-exclusive detections in the 3D system (Figure 3a). Correlation analysis of shared phosphopeptides revealed a positive but limited relationship between 2D neurons and 3D COs (r = 0.597; Figure 3b). Unlike the global proteome, phosphoproteomic profiles showed greater variability, with within-model pairwise Pearson correlations ranging from approximately r = 0.6–0.7 for both 2D and 3D models. Cross-model correlations were consistently lower, at approximately r = 0.5 (Supplementary Figure S2a). A similar correlation was observed at the phosphoprotein level (r = 0.552; Supplementary Figure S2b), supporting that phosphorylation is highly context-dependent. This lower reproducibility compared with total proteome measurements is consistent with the rapid and reversible nature of phosphorylation (Cohen, 2002; Trinidad et al., 2012), which is dynamically regulated by cellular signaling.

**FIGURE 3:**
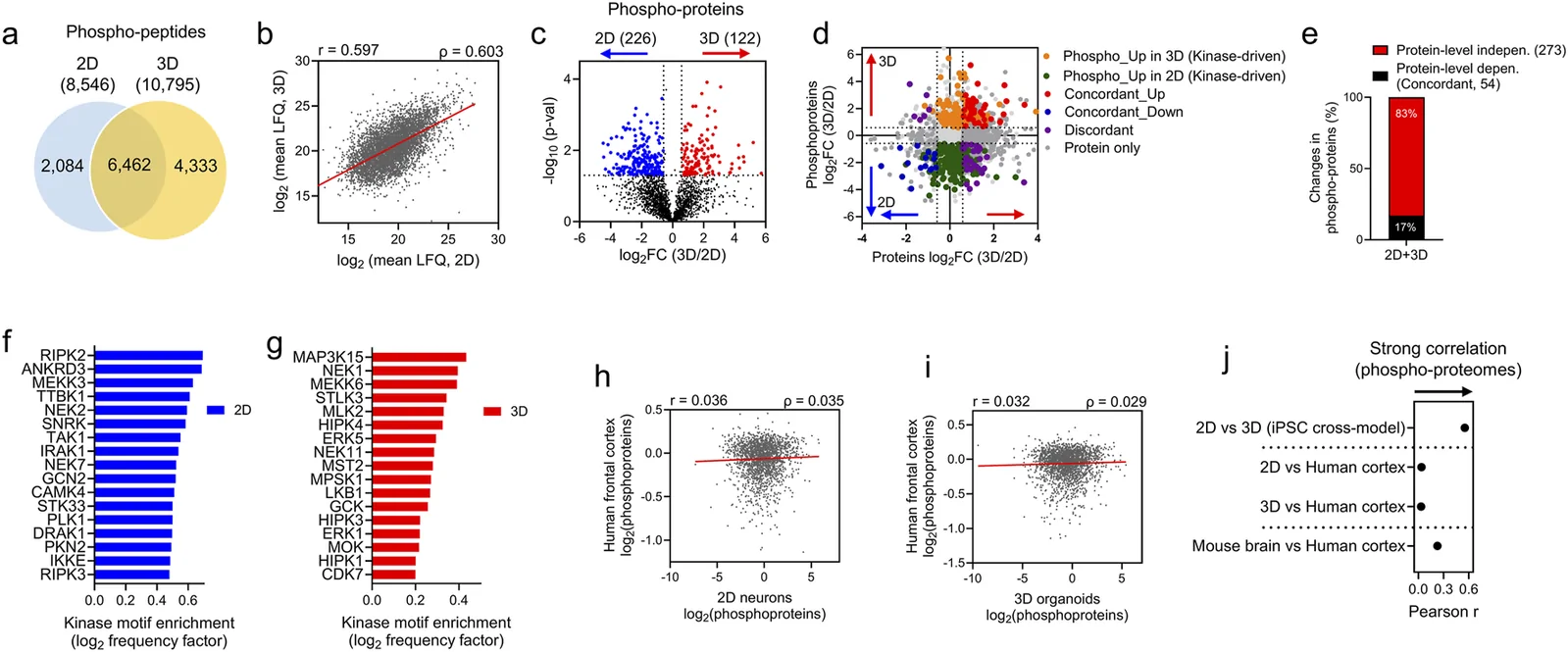
Comparative phosphoproteomic profiling of 2D neurons and 3D cortical organoids. **(a)** Venn diagram showing the number of phosphopeptides identified in 2D and 3D, including shared and model-specific phosphopeptides. **(b)** Scatter plot showing the correlation of phosphopeptide abundance between 2D and 3D for shared phosphopeptides. **(c)** Volcano plot showing differentially abundant phosphopeptides between 2D and 3D. Phosphopeptides enriched in 2D and 3D are indicated in blue and red, respectively. **(d)** Integration of phosphoproteomic data with global protein abundance to dissect whether changes in phosphoprotein levels are driven by alterations in protein expression levels. The scatter plot separates protein abundance-driven phosphoproteomic changes from protein-level-independent changes that may reflect kinase- or phosphatase-mediated regulation. **(e)** Stacked bar plot summarizing the proportion of differentially abundant phosphoproteins classified as protein-level dependent (concordant, 54 phosphoproteins) or protein-level independent (273 phosphoproteins). The high proportion of protein-level-independent changes suggests that phosphoproteomic remodeling is predominantly driven by altered signaling activity rather than changes in total protein abundance. **(f,g)** Kinase motif enrichment analysis of phosphopeptides enriched in 2D neurons **(f)** and 3D cortical organoids **(g)**. **(h,i)** Scatter plots showing the correlation between the published human frontal cortex phosphoproteome and our 2D neuron phosphoproteome **(h)** or 3D CO phosphoproteome **(i)**. **(j)** Summary of Pearson correlation coefficients comparing 2D and 3D phosphoproteomes with published reference phosphoproteomes. Higher Pearson’s r values indicate greater phosphoproteomic similarity.

Volcano plot analysis further identified distinct sets of differentially abundant phosphoproteins enriched in each model, demonstrating that 2D neurons and 3D organoids have divergent phosphorylation landscapes (Figure 3c). To determine whether changes in phosphorylation were driven by altered total protein abundance or by phosphorylation-specific regulation, we integrated phosphoproteomic data with matched global protein abundance profiles (Figure 3d). Integration analysis separated protein abundance-driven phosphorylation changes from protein-level-independent changes, which are mainly mediated by altered kinase or phosphatase activity. Differentially abundant phosphoproteins were divided into protein-level-dependent changes and protein-level-independent changes. Only 54 phosphoproteins showed concordant phosphorylation changes with total protein abundance, whereas 273 phosphoproteins were classified as phosphorylation-specific or discordant with protein-level changes (Figure 3e). Using the present statistical criteria, 83% of nominally altered phosphoproteomic signals were not accompanied by an FDR-significant change in the corresponding total protein (Figure 3e). Although 2D neurons and 3D COs share many phosphopeptides, phosphorylation landscapes differ substantially between 2D and 3D, showing a stronger contribution from protein-level-independent signaling changes.

Kinase motif enrichment analysis revealed model-specific signaling programs. In 2D neurons, motifs associated with RIPK2, ANKRD3, MEKK3, and TTBK1 showed higher enrichment. In contrast, 3D COs showed increased enrichment of motifs associated with MAP3K15, MEKK6, STLK3, MLK2, and HIPK4, suggesting enhanced activation of stress-responsive MAPK-related upstream regulatory pathways in the 3D model (Figures 3f,g). Kinase-substrate enrichment analysis (KSEA) further supported distinct signaling states between the two models. The dominant kinase activity signature in 3D organoids involved increased PPP1CB, PTK2, PDK1, and RICTOR/mTORC2 signaling, consistent with extracellular matrix engagement, 3D tissue architecture, and integrin-associated signaling. By contrast, tyrosine phosphatase-related signatures, including PTPRJ, were relatively increased in 2D neurons, consistent with suppression of EGFR, MAPK, and SRC-related phosphorylation pathways (Supplementary Figure S2c). Together, these findings indicate that 3D organoids exhibit a more signaling-active phosphoproteomic state, whereas 2D neurons display a comparatively distinct phosphorylation profile shaped by monolayer culture conditions.

To benchmark phosphoproteomic profiles against human brain tissue, we compared gene-level phosphoprotein abundance in 2D and 3D models against a published human adult frontal cortex phosphoproteome (Ping et al., 2020) (syn20820053). Neither 2D neurons nor 3D COs showed measurable correlation with the adult human frontal cortex phosphoproteomic reference (r = 0.036 and r = 0.032, respectively; Figures 3h,i), demonstrating that phosphoproteomic profiles are highly sensitive to differences in developmental stage, tissue context, and cellular composition. Summary correlation analysis across reference phosphoproteomic datasets further confirmed our data: while 2D and 3D phosphoproteomes were moderately correlated with each other (r ≈ 0.6), both showed no correlation with adult human cortex phosphoproteomes (Figure 3j). Notably, mouse whole-brain phosphoproteome similarly showed very weak correlation with human adult frontal cortex phosphoproteome (r ≈ 0.23), indicating that the absence of phosphoproteomic similarity between iPSC-derived 2D/3D models and adult human cortex reflects a fundamental developmental and species-level divergence in phosphorylation signaling rather than a limitation of *in vitro* models. Our data establish phosphoproteomics as a sensitive benchmark for developmental stage, signaling context, and disease conditions, highlighting the advantages of paired global proteomic and phosphoproteomic profiling for studying the pathophysiology of human neurodevelopmental disorders.

## Discussion

In this study, we performed paired global proteomic and phosphoproteomic profiling of human iPSC-derived 2D cortical neurons and 3D brain COs, and systematically benchmarked both models against published human and mouse brain reference datasets. Our data demonstrate that both 2D and 3D models show fetal brain-like proteomic similarity and diverge substantially from adult human brain at the phosphoproteomic level. Together, these results provide a molecular framework for evaluating the fidelity of human neural models and reveal fundamentally different conservation properties of the proteome and phosphoproteome across developmental stages, species, and experimental contexts.

Both 2D cortical neurons and 3D COs showed higher proteomic correlation with human fetal brain than with mouse cerebral cortex, consistent with prior transcriptomic studies demonstrating that iPSC-derived neural models capture early developmental gene expression programs (Quadrato et al., 2017). This finding is particularly notable given that the reference datasets were generated using different mass spectrometry platforms and quantification strategies, suggesting that the proteomic similarity reflects genuine biological correspondence rather than technical artifact. The higher correlation with fetal brain than with adult cortex is expected, as iPSC-derived neurons and organoids represent an immature developmental state comparable to second-trimester human cortex (Camp et al., 2015; Pollen et al., 2019). The fact that both 2D and 3D models showed proteomic correlations with human fetal brain comparable to inter-study variability among independent fetal brain datasets underscores the validity of 2D and 3D as human neurodevelopmental models. In contrast, mouse cerebral cortex proteome showed lower correlation with human adult cortex (r ≈ 0.55), consistent with prior reports of substantial cross-species divergence in brain protein expression (Zhu et al., 2018), supporting the unique value of human iPSC-based systems for modeling human-specific neurodevelopmental biology.

Our findings reveal that both 2D and 3D models show fetal-brain-biased global proteomic similarity. However, this similarity should not be interpreted as recapitulation of a defined fetal cortical state or correspondence to a specific gestational age. The fetal cortex comprises dynamic and overlapping developmental programs rather than a single molecular state (Micali et al., 2023; Nowakowski et al., 2017; Wang et al., 2025). Both 2D and 3D models expressed proteins associated with radial glia and neural progenitors (SOX2, VIM, NES, and EOMES), early deep-layer neurogenesis (TBR1 and BCL11B), later upper-layer neurogenesis (SATB2 and CUX1), and gliogenic lineages (GFAP, S100B, and AQP4). These marker-based observations provide biological context for fetal-brain-biased proteomic similarity, but do not establish equivalence to a defined fetal cortical stage.

Despite sharing a broad neural proteomic background, 2D neurons and 3D COs exhibited model-specific protein abundance differences, which likely reflect the distinct cellular compositions, culture architectures, and maturation states of the two systems. 3D COs contain a heterogeneous mixture of neural progenitors, cortical excitatory neurons, and astroglia-like cells organized in spatial proximity, whereas 2D cultures are enriched for post-mitotic cortical neurons in a simplified monolayer (Lancaster et al., 2013; Sloan et al., 2018). The two models thus provide complementary rather than redundant proteomic coverage: 2D neurons offer a defined and reproducible cellular context well-suited to mechanistic and pharmacological studies, while 3D COs better capture aspects of multicellular organization and developmental heterogeneity relevant to *in vivo* brain tissue.

The 3D cultures expressed neuronal and cortical-lineage markers and exhibited spontaneous electrophysiological activity on MEA, demonstrating the functional activity of the organoids. However, these data do not establish ventricular-zone organization, radial migration, or layer-specific spatial organization; therefore, we conservatively designate our 3D cultures as cortical-patterned neural organoids. Moreover, because bulk proteomic analysis cannot distinguish differences in cell-type abundance from cell-intrinsic regulation, the enrichment of progenitor-, ECM-, and glial-associated proteins may partly reflect compositional differences between the 2D and 3D cultures rather than effects intrinsic to three-dimensional culture alone (Benito-Kwiecinski et al., 2021).

The most striking finding of this study is the near-complete absence of phosphoproteomic correlation between iPSC-derived models and adult human frontal cortex reference datasets. While this divergence is partly attributable to the developmental immaturity of iPSC-derived neurons relative to adult brain tissue, an equally important and frequently underappreciated confound is the instability of the phosphoproteome in postmortem human tissue. Protein phosphorylation is an intrinsically labile modification, rapidly altered by agonal state, postmortem interval, tissue pH, and freeze-thaw cycles (Li et al., 2003; Wang et al., 2015). Postmortem phosphoproteomic datasets, including the Ping et al. (2020) (Ping et al., 2020) dataset used as a reference in this study, are therefore likely to reflect a substantially degraded phosphorylation landscape relative to the *in vivo* physiological state. In contrast, iPSC-derived neurons are harvested under controlled live-cell conditions, preserving phosphorylation states that more closely reflect active kinase and phosphatase signaling. The comparison between a postmortem-derived adult cortex phosphoproteome and a live-cell iPSC phosphoproteome is therefore confounded by both developmental stage and tissue preservation state, thereby supporting the near-zero correlation. This interpretation is further supported by the observation that even mouse whole-brain phosphoproteome showed only minimal correlation with human adult frontal cortex phosphoproteome (r ≈ 0.23), despite comparable neural tissue identity, suggesting that phosphoproteomic profiles are globally sensitive to species, developmental stage, experimental platform, disease conditions, and postmortem tissue quality. Unlike the proteome, which reflects relatively stable cellular composition and neural identity, the phosphoproteome recapitulates dynamic kinase-mediated signaling states that are exquisitely sensitive to cellular context and disease conditions. This highly context-dependent nature of the phosphoproteome has important implications for modeling neurodevelopmental disorders.

Several limitations should be acknowledged. First, the human adult frontal cortex phosphoproteomic reference (Ping et al., 2020) was derived from postmortem tissue with variable postmortem intervals, which likely contributes to the low observed correlation with iPSC-derived model phosphoproteomes independent of developmental stage differences. Brain phosphorylation is known to change rapidly after death (Li et al., 2003). The absence of a comparable human fetal phosphoproteomic reference dataset prevents us from determining whether the phosphorylation profiles of the 2D and 3D models recapitulate a fetal developmental state. Second, public reference datasets were generated using different mass spectrometry platforms, sample preparation methods, enrichment strategies, and quantification approaches, which may reduce cross-study correlations independently of biological differences. In addition, the near-zero correlations between the *in vitro* phosphoproteomic profiles and the adult frontal-cortex phosphoproteomic reference might be interpreted as a technically constrained cross-study observation rather than as evidence of developmental or species-related divergence. The datasets differ in sample source, preservation, cellular composition, postmortem interval, quantitative strategy (TMT versus LFQ), analytical platform, and phosphosite coverage (Ping et al., 2020). Third, gene-level aggregation of phosphopeptides improves cross-dataset comparability but masks site-specific phosphorylation changes and stoichiometry. Aggregation of phosphopeptide measurements at the gene level facilitated cross-dataset integration but resulted in the loss of site-specific biological information. Because individual phosphorylation sites within the same protein may be regulated by different kinases and have distinct or opposing functional effects, gene-level changes should not be interpreted as evidence of specific phosphorylation mechanisms. Future site-resolved analyses and targeted validation will be required to determine the functional consequences of individual phosphosite changes. KSEA and motif-enrichment analyses identified differences in inferred kinase-associated signatures between the 2D and 3D models. Because KSEA and motif-enrichment analyses infer upstream regulation from the detected phosphosite patterns and are influenced by substrate coverage, the underlying knowledge base, and the analytical algorithm, these analysis do not directly measure kinase expression or activation. Accordingly, the PTK2-, PDK1-, and RICTOR/mTORC2-associated signals are presented as exploratory candidates rather than evidence of established kinase-specific mechanisms. Fourth, bulk proteomic and phosphoproteomic profiling does not resolve cell-type-specific signals within the heterogeneous cellular populations of 3D COs; future single-cell or cell-type-sorted proteomics would provide higher resolution. Finally, this study used a single control iPSC line; replication across multiple donor lines will be necessary to assess the generalizability of the observed proteomic and phosphoproteomic profiles. Although using the same iPSC line enabled a controlled comparison, validation across multiple donor lines is needed to account for donor-specific variation and establish the generalizability of our findings. Statistical power of the phosphoproteomic analysis is limited, because the 2D group included only two biological replicates and no individual phosphoproteins remained significant after FDR correction. Accordingly, phosphoproteomic profiles should be considered exploratory and hypothesis-generating rather than evidence of established model-specific phosphorylation mechanisms. The absence of validation by methods such as Western blotting or immunostaining limits confidence in individual protein and phosphoprotein changes, which should be considered exploratory candidates for future validation.

Additionally, an important limitation of this study is that the 2D cultures and 3D organoids were analyzed at different differentiation time points. These time points represent protocol-specific endpoints at which neuronal maturation and glial development are reliably established, rather than chronologically or developmentally equivalent stages. Therefore, the observed enrichment of synaptic, calcium-signaling, extracellular-matrix, and proteostasis-related proteins in day-110 organoids cannot be attributed specifically to 3D architecture, as it may also reflect differences in culture duration, developmental stage, cellular composition, and differentiation protocol (Scuderi et al., 2021). Accordingly, our findings should be interpreted as a comparison between two distinct culture states rather than as evidence of a causal 2D-versus-3D effect. Nevertheless, both models showed the strongest proteomic similarity to the human fetal cortex, supporting complementary platforms for studying early human cortical development. Each system offers distinct advantages and limitations, and combined use of 2D and 3D models may provide a more comprehensive representation of human neurodevelopment.

The paired proteomic and phosphoproteomic benchmarking framework developed in our study could be extended to disease-specific iPSC-derived neuronal and organoid models. In neurodevelopmental disorders such as autism spectrum disorder (ASD), longitudinal, stage-matched comparisons with control or isogenic lines may reveal alterations in differentiation trajectories, neuronal subtype specification, synaptic maturation, and phosphorylation-associated signaling. Similarly, in neurodegenerative disorders such as Alzheimer’s disease, this approach could characterize progressive molecular changes across aging, stress, or degeneration paradigms. Integration with functional assays and therapeutic screening may further identify interventions capable of restoring disease-associated molecular and cellular phenotypes.

In conclusion, human iPSC-derived 2D cortical neurons and 3D COs show fetal brain-like proteomic profiles that more closely resemble human brain tissue than mouse cortex, supporting 2D and 3D as human-relevant neurodevelopmental models. The phosphoproteome, by contrast, is highly sensitive to developmental stage, tissue context, and pathological conditions, making it valuable for functional cellular state rather than neural identity *per se*. Together, our data establish paired global proteomic and phosphoproteomic profiling as a powerful analytical framework for evaluating the molecular fidelity of human neural models, and highlight the complementary strengths of 2D neuronal cultures and 3D COs for studying neurodevelopmental disorder pathophysiology and for screening therapeutic interventions that target disease-relevant protein abundance and signaling states.

## Data Availability

All analysis scripts are deposited at GitHub (https://github.com/yongsoopark01/ipsc-neuron-organoid-proteomics) and archived on Zenodo (DOI: 10.5281/zenodo.20176358). Processed LFQ protein and phosphopeptide intensity data are deposited at Zenodo (DOI: 10.5281/zenodo.20176759).

## Supplementary material

SUPPLEMENTARY FIGURE S1 Global proteomic comparison of 2D neurons and 3D cortical organoids. **(a)** Principal component analysis (PCA) of global proteomic profiles from 2D neurons and 3D cortical organoids, showing distinct separation between the two models based on protein abundance patterns. **(b)** Pearson correlation matrix showing pairwise correlations among biological replicates within each model and across models. Higher correlation values indicate greater similarity in global protein abundance profiles. **(c,d)** Gene Ontology (GO) enrichment analysis of proteins enriched in 2D neurons **(c)** and 3D cortical organoids **(d)**, highlighting biological processes and functional pathways associated with each model.

SUPPLEMENTARY FIGURE S2 Comparative phosphoproteomic analysis of 2D neurons and 3D cortical organoids. **(a)** Pearson correlation matrix showing pairwise correlations of phosphoproteomes among biological replicates within and across models. The two 2D samples were generated from independent differentiation experiments, whereas the 3D samples comprised individual organoids maintained in separate wells but derived from a single differentiation batch. **(b)** Scatter plot showing the correlation of phosphoprotein abundance between 2D neurons and 3D cortical organoids, with a Pearson correlation coefficient of r = 0.552. **(c)** Kinase-substrate enrichment analysis (KSEA) comparing predicted kinase activity patterns between 2D neurons and 3D cortical organoids. Kinases enriched in 2D neurons are indicated in blue, whereas kinases enriched in 3D cortical organoids are indicated in red.
