# Supplementary material for: Human iPSC-derived 2D cortical neurons and 3D cortical organoids show fetal brain proteomic similarity and distinct phosphoproteomic profiles
Source: Front Bioeng Biotechnol. 2026 Sep 24;14:1932407. doi: 10.3389/fbioe.2026.1932407 (PMC13646154; doi:10.3389/fbioe.2026.1932407)

Supplementary Figure 1

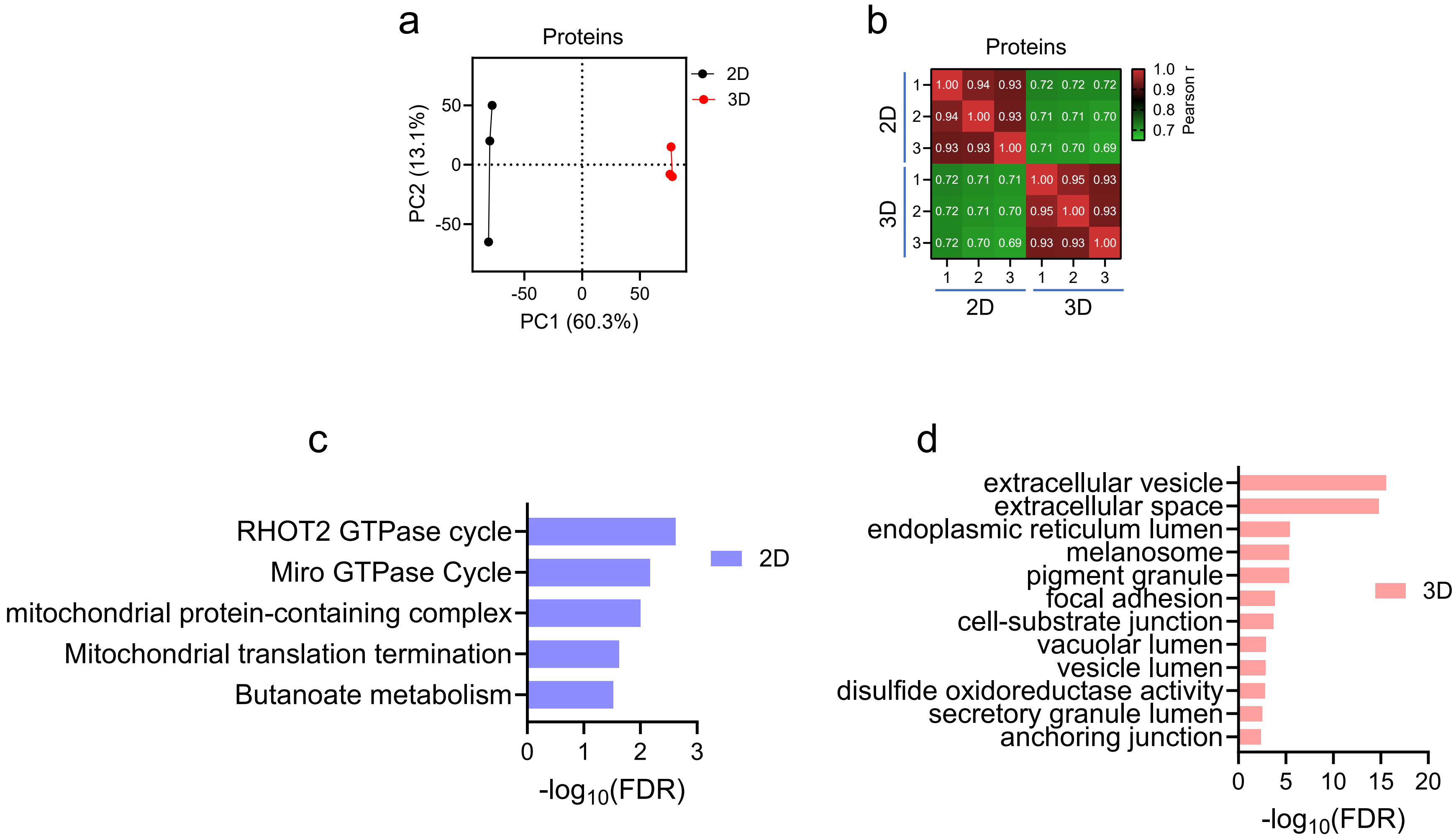

Supplement: Supplementary file 1 [file Supplementaryfile1.pdf]
